# Effectiveness of a digital intervention versus alcohol information for online help-seekers in Sweden: a randomised controlled trial

**DOI:** 10.1186/s12916-022-02374-5

**Published:** 2022-05-17

**Authors:** Marcus Bendtsen, Katarina Åsberg, Jim McCambridge

**Affiliations:** 1 Department of Health, Medicine and Caring Sciences, Division of Society and Health, Linköping University, 581 83 Linköping, Sweden; 2 Department of Health Sciences, University of York, York, England

**Keywords:** Brief alcohol intervention, Digital behaviour change intervention, Public health, Telemedicine, Randomised controlled trial

## Abstract

**Background:**

The ubiquity of Internet connectivity, and widespread unmet needs, requires investigations of digital interventions for people seeking help with their drinking. The objective of this study was to test the effectiveness of a digital alcohol intervention compared to existing online resources for help seekers.

**Methods:**

This parallel randomised controlled trial included 2129 risky drinkers with access to a mobile phone and aged 18 years or older. Randomised sub-studies investigated consent procedures and control group design. Simple computerised randomisation was used. Participants were aware of allocation after randomisation; research personnel were not. The digital intervention was designed around weekly monitoring of alcohol consumption followed by feedback and tools for behaviour change. Primary outcomes were total weekly consumption (TWC) and frequency of heavy episodic drinking (HED), measured 2 and 4 months post-randomisation.

**Results:**

Between 25/04/2019 and 26/11/2020, 2129 participants were randomised (intervention: 1063, control: 1066). Negative binomial regression was used to contrast groups, with both Bayesian and maximum likelihood inference. The posterior median incidence rate ratio (IRR) of TWC was 0.89 (95% CI = 0.81;0.99, 98.2% probability of effect, *P*-value = 0.033) at 2 months among 1557 participants and 0.77 (95% CI = 0.69;0.86, > 99.9% probability of effect, *P*-value < 0.001) at 4 months among 1429 participants. For HED, the IRR was 0.83 (95% CI = 0.75;0.93, > 99.9% probability of effect, *P*-value = 0.0009) at 2 months among 1548 participants and 0.71 (95% CI = 0.63;0.79, probability of effect > 99.9%, *P*-value < 0.0001) at 4 months among 1424 participants. Analyses with imputed data were not markedly different.

**Conclusions:**

A digital alcohol intervention produced self-reported behaviour change among online help seekers in the general population. The internal and external validity of this trial is strong, subject to carefully considered study limitations arguably inherent to trials of this nature. Limitations include higher than anticipated attrition to follow-up and lack of blinding.

**Trial registration:**

The trial was prospectively registered (ISRCTN48317451).

**Supplementary Information:**

The online version contains supplementary material available at 10.1186/s12916-022-02374-5.

## Background

Despite clear evidence of the risks of consuming alcohol, both drinking and heavy drinking continue to be highly prevalent and socially acceptable in many societies [1]. Being produced and sold legally does not detract from the fact that alcohol is an addictive drug which causes a great deal of harm [2]. Alcohol consumption has been found to increase the risk of non-communicable diseases, including stroke, heart failure, and cancer, and there is no safe dose [1, 3]. Overall, alcohol has been estimated to contribute to approximately 2.6% of disability-adjusted life years (DALYs) among women and 7.5% of DALYs among men world-wide [1]. In addition to the risks to the health of the drinker, alcohol consumption causes harms to others and to society, including traffic accidents, violence, fetal damage, and harms to family members, as well as placing avoidable burdens on health, criminal justice, and welfare systems [4–6].

With the ubiquity of Internet connectivity in high-income countries, and increasingly in low- and middle-income countries, an obvious way to extend reach into the community is by offering *digital* alcohol interventions. Such interventions have been found to be effective in helping individuals to reduce their alcohol consumption in a range of populations and settings. A meta-analysis of digital interventions suggested that unguided interventions to non-student populations may reduce total weekly consumption by 32.3 g (95% CI = 5.9;58.8) [7]. A Cochrane Review of trials including both student and non-student populations found that digital interventions may reduce total weekly consumption by 22.8 g (95% CI = 15.4;30.3) and also that the frequency of heavy episodic drinking may be reduced by 0.24 episodes per month (95% CI = 0.13;0.35) [8]. Finally, a meta-analysis of text messaging interventions found that these interventions may reduce total weekly consumption by 18.6 g (95% CI = −2.38;39.6) and frequency of heavy episodic drinking by 0.33 episodes per month (95% CI = −0.12;0.79) [9]. While these findings are encouraging, all three meta-analyses found issues with heterogeneity and high risk of bias in many of the included trials. Thus, the synthesised body of evidence supporting the use of digital alcohol interventions is not without issue.

Variability in the effects of these kinds of interventions in different cultural contexts and populations may be expected [10–12], though this has not been well studied. Evidence on detailed content, mechanisms of action, and mediators of effects of digital interventions is limited, perhaps unsurprisingly, as this is the case with brief alcohol interventions more broadly [13]. So, while offering digital alcohol interventions to those seeking help online is attractive for reach purposes, there are many uncertainties about how to do so most effectively [14–16].

This study aimed to investigate if a digital alcohol intervention designed around weekly monitoring of consumption followed by feedback and tools for behaviour change can help reduce both total weekly consumption and frequency of heavy episodic drinking among people seeking help online in Sweden. A secondary aim of the study was to estimate the effects of the intervention on risky drinking.

## Methods

### Study design

A 2-arm parallel group randomised controlled trial with simple randomisation was employed to study the effects of a digital alcohol intervention in contrast to an alcohol information control. Nested within the trial were two sub-studies which explored (1) whether different layouts of the informed consent materials affected consent rates and recall of trial procedures and (2) whether variations in information content affected rates at which further information was accessed. There were no deviations from the study protocol [17] after trial commencement. The trial was prospectively registered (ISRCTN48317451) and received ethical approval on 06/11/2018 by the Regional Ethical Committee in Linköping, Sweden (Dnr 2018/417-31).

### Participants

The target population was Swedish adults seeking help online to reduce their alcohol consumption. Individuals were required to be at least 18 years of age, have access to a mobile phone, and be classified as risky drinkers according to Swedish guidelines. This is defined as either drinking 9 (women)/14 (men) or more standard drinks of alcohol per week (total weekly consumption) or drinking 4 (women)/5 (men) or more standard drinks on a single occasion at least once a month (heavy episodic drinking). A standard drink is in Sweden defined as 12 g of alcohol. All study materials were in Swedish, which meant that individuals who did not comprehend Swedish well enough to understand these were implicitly excluded.

**Fig. 1:**
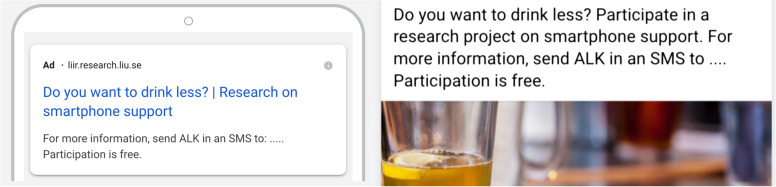
Example adverts used on Google, Bing, Yahoo, and Facebook (translated to English)

Participants were recruited to the trial using web search engine advertisements (Google, Yahoo, Bing) and Facebook. Examples of advertisements used are shown in Fig. 1 (translated to English). Individuals interested in the study sent a text message to a dedicated phone number. Within 10 min, a response was sent back with a hyperlink to a web page which presented the informed consent material.

Those who consented were asked to respond to a baseline questionnaire (which also assessed eligibility). The questionnaire included questions on demographics, current alcohol consumption, and three single-item measures of confidence in one’s ability to reduce drinking, perceived importance of reducing drinking, and knowledge of how to reduce drinking. Please see Additional file 2 for full details on questions asked at baseline. Eligible participants were randomised immediately after responding to the baseline questionnaire.

### Randomisation and masking

We used simple randomisation which was fully computerised. No blocks or strata were employed. Once the baseline questionnaire was completed, each eligible participant was randomised by the backend server and allocation was done automatically. Neither participants nor research personnel were able to discover or in any way manipulate the randomisation sequence.

Research personnel were blinded before and after allocation, and all study procedures were fully automated, except for the follow-up phone calls, where needed. There was some risk of allocation being revealed to personnel during follow-up calls (please see the “Limitations” section). Participants were not blind after allocation, as they were aware if they received immediate access to the digital intervention or not.

### Procedures

#### Consent

Individuals were randomised to two different layouts of the informed consent materials (the first sub-study of the trial). One of the layouts (Consent-1) showed all consent materials immediately on the web page, while the other layout required that participants click on hyperlinks for parts of the information (Consent-2). No information was withheld from either group; the experimental contrast lay only in how the information was presented. Please see Additional file 1 for the material and further explanation of the two different layouts.

Although it may appear easy to obtain informed consent in online trials, the extent to which consent is truly informed is questionable. Challenges include conveying that interventions studied are yet to be shown effective, as well as the concept of control groups and randomised allocation [18]. Experiments examining length and visual variations of informed consent materials have explored comprehension and individual preferences [19, 20]; however, consent rates and recall of study procedures due to layout differences have not been explored in a naturalistic setting. This trial allowed for a sub-study to be included without any interference with other study procedures, thus allowing for a naturalistic evaluation of how changes to the layout of informed consent materials affect consent rates and recall of trial procedures in online settings. It was not possible to obtain informed consent from participants for this sub-study, as it would have invalidated the experiment. Analyses of recall will be presented separately; here, we report on consent rates and effects on primary outcomes.

#### Digital intervention

The core element of the digital intervention was a text message sent to participants each Sunday afternoon. The text message included a prompt to self-monitor one’s current alcohol consumption, with a hyperlink to a web-based tool. Those who decided to click on the link were given access to a personalised support tool after providing data. Please see Additional file 3 [21–24] for more information on the intervention.

Participants were instructed to send a text message with the word *stop* if they no longer wish to receive any more text messages (including weekly assessments). Access to the intervention was restricted to 4 months; however, it should be noted that this restriction was purely for research purposes. In a non-research setting, individuals would be able to engage with this intervention for as long as they found it helpful.

#### Alcohol information

Participants allocated to the control group were advised that they would receive information designed to motivate them to think more about reducing their alcohol consumption and that after 4 months they would receive additional support delivered to their mobile phone. Thus, individuals allocated to the control setting were given access to the digital intervention after completion of the final follow-up.

The control group was further randomised into two groups (the second sub-study of the trial). Participants in both control groups received a single text message with basic health information regarding short- and long-term effects of alcohol consumption. However, we incorporated a contrast between two very brief types of information: one which emphasised possible complexities associated with the short- and long-term effects of alcohol (such as is widely available from alcohol industry sources, Info-1) and another which provided a clear and straightforward public health messaging style (while being appropriately evidence informed, Info-2). Each message was delivered in a single text and included the same link to a website with information about alcohol (https://www.iq.se). Please see Additional file 4 for the full content of both text messages.

The effect of an intervention should always be understood as a contrast relative to a control condition [25]; thus, understanding the control condition is key for interpreting estimated effect sizes. Despite this, attention to the design of control conditions is underdeveloped [26–29]. It is common to use basic health information as a control condition in behavioural intervention trials, and much information is available online of variable quality. There is, however, little actual study evaluating the effects of widely available alcohol or other health information. The sub-study included in this trial allowed us to explore the effects of being exposed to very brief alcohol information with different contents on the rate at which more information was requested (i.e. clicking on the supplied link) and alcohol consumption (primary outcomes). Thus, this sub-study aimed to assist further consideration of the design of control conditions [30]. As individuals who enrolled were looking for help to change their alcohol consumption, we anticipated that most participants would be motivated to click on the link. It was however considered plausible that either type of message would encourage participants more than the other to click on the link. We compared two types of information as a preliminary study: standard public health and alcohol industry-generated material. It was considered plausible that participants who received the alcohol industry worded message where risks related to alcohol are downplayed and portrayed as complex would be satisfied that their current drinking behaviour was not strongly linked to health issues and therefore less interested in accessing more information. Alternatively, the suggestion of complexity may have motivated curiosity.

As there was some information provided to all participants (including those who did not click on the included link), we refer to the control condition as alcohol information.

### Outcomes

#### Measures

Primary

- Total weekly alcohol consumption
- Frequency of heavy episodic drinking

Secondary

- Classification as a risky drinker according to Swedish guidelines

Total weekly alcohol consumption was measured using a short-term recall method [31] by asking participants the number of standard drinks consumed the past week. Using a summary measure, rather than asking day-by-day, allowed for the same question to be asked regardless of whether responses were collected via web questionnaire, text message, or phone interviews (see the “Follow-up” section). The frequency of heavy episodic drinking was assessed by asking participants how many times they consumed 4 (women)/5 (men) or more standard drinks on one occasion in the past month. Classification as a risky drinker was calculated based on responses to the two primary outcome measures.

#### Follow-up

Primary and secondary outcomes were assessed at 2 and 4 months post-randomisation. We also conducted a 1-month follow-up which assessed confidence, importance, and knowledge, which were used for planned mediator analyses (reported separately).

All follow-ups were initiated by sending text messages to participants with hyperlinks to web questionnaires. A total of two reminders were sent 2 days apart to those who had not responded. If no response was collected after the second reminder, a fourth text message was sent to participants asking them to respond to the two primary outcome measures by responding directly with a text. We called participants to collect responses if there was no response to the fourth text message (maximum of five calls).

### Statistical analysis

#### Sample size

The required sample size was determined using Monte Carlo simulations. A full description of the simulations is available in the study protocol [17]; thus, for succinctness, we restrict the description here to the most relevant parts.

We believed that a minimal relevant effect for the type of intervention studied, taking into consideration the unguided nature of the intervention and the setting, would be if the intervention group was consuming 15% less alcohol per week at the 4-month follow-up in comparison to the control group. We aimed for an expected power of 80% at the 0.05 significance threshold. Based on our previous studies of digital interventions in Sweden [32, 33], we expected an attrition rate between 5 and 25%. The simulations suggested an expected sample size of 2126 individuals (interquartile range = 2031;2198).

Participants were recruited over a series of 6-month periods. Between each period, we checked if the planned sample size had been achieved. Between 25/04/2019 and 26/11/2020, we randomised 2129 participants, at which time recruitment was stopped. This equates to approximately 19 months of recruitment, having allowed an initial grace period of 1 month for advert placement algorithms to optimise their performance.

#### Primary and secondary outcomes

All individuals were analysed in the groups to which they were randomised (intention-to-treat). Missing data was initially handled by complete-case analyses, and sensitivity analyses were performed with missing data imputed (using multiple imputation by chained equations). All analyses were done using R version 4.05 with packages: rstan version 2.21.2, mice version 3.13.0, and MASS version 7.3.54.

Regression models were estimated using both Bayesian inference and maximum likelihood estimation (MLE), and both methods were used for scientific inference [34]. The medians of posterior distributions were taken as point estimates, with 95% credible intervals (CI). Null hypothesis testing of MLE estimates was done at the 0.05 significance level (two-tailed).

Total weekly alcohol consumption and heavy episodic drinking (primary) were analysed using negative binomial regression, and classification as a risky drinker (secondary) was analysed using logistic regression. Both unadjusted and adjusted models were estimated, with adjusted models being primary. As specified in the protocol [17], adjusted models included covariates for baseline values of the respective primary outcome, sex, civil status, age, motivation, importance, and knowledge.

We estimated interaction models for each primary outcome and each baseline characteristic respectively. We also estimated multi-level models for the primary outcomes with random intercept and slope for age, as drinking varies among age groups. The models were compared to the primary adjusted models using the Widely Applicable Information Criterion (WAIC) and likelihood ratio tests.

#### Attrition analyses

Attrition analyses investigated if responders and non-responders differed systematically with respect to baseline characteristics, and among study groups, for which we used logistic regression estimated with Bayesian inference with shrinkage priors [35] to account for the excessive number of covariates.

Based on the assumptions of repeated attempt models [36, 37], a second analysis investigated if late responders to follow-up were more like non-responders than early responders to follow-up. An association between attempts to collect follow-up and outcomes could in such a case imply systematic differences between non-responders and responders. To explore this assumption, the primary outcomes were regressed against follow-up attempt with an interaction for group allocation and adjusted for each respective outcome measure at baseline (negative binomial regression with shrinkage priors).

## Results

**Fig. 2:**
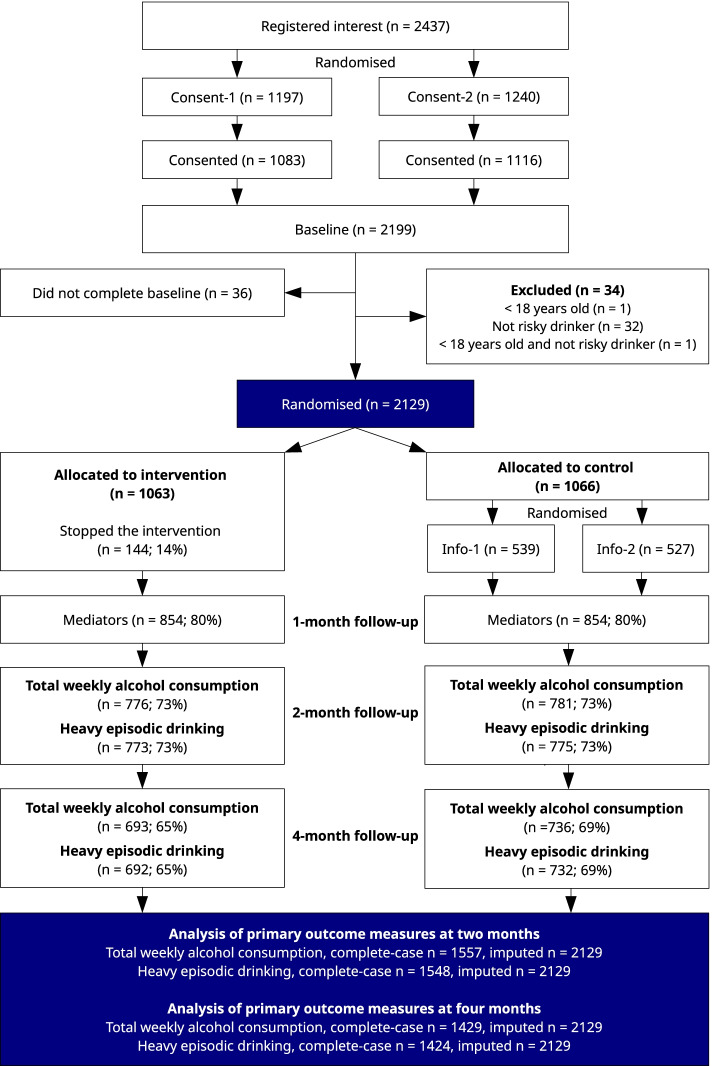
Participant flow presented in a CONSORT flow diagram

A diagram describing participant flow is presented in Fig. 2. Between 25/04/2019 and 26/11/2020, 2437 individuals registered interest in the trial and were randomised to either of the two layouts of informed consent materials (Consent-1 and Consent-2). In total, 2199 participants consented (90.2% of registered participants), and there was evidence of little difference in odds of consent given the two layouts (OR = 1.06, 95% CI = 0.81;1.37, probability of effect = 65.6%, *P*-value = 0.69).

There were 36 consenting participants who did not complete the baseline questionnaire and 34 who were excluded due to not fulfilling the inclusion criteria. The remaining 2129 participants were randomised: 1063 to the intervention group and 1066 to the control group. Baseline characteristics of the randomised participants were well distributed; see Table 1.

**Table 1:** Baseline characteristics of randomised participants

	Total (***n*** = 2129)	Intervention (***n*** = 1063)	Control (***n*** = 1066)
Total weekly alcohol consumption, median (quartiles)	17 (10;25)	17 (10;25)	16 (10;25)
Frequency of heavy episodic drinking, median (quartiles)	6 (4;11)	6 (4;10)	6 (4;12)
Risky drinking	2129 (100%)	1063 (100%)	1066 (100%)
Age, median years (quartiles)	45 (36;54)	45 (35;55)	46 (36;54)
Sex, *n* (%)			
Women	1237 (58%)	612 (58%)	625 (59%)
Men	892 (42%)	451 (42%)	441 (41%)
Civil status, *n* (%)			
Living alone without kids at home	443 (21%)	219 (21%)	224 (21%)
Living alone with kids at home	215 (10%)	114 (11%)	101 (9%)
Living with somebody without kids	544 (26%)	267 (25%)	277 (26%)
Living with somebody with kids	756 (36%)	383 (36%)	373 (35%)
Have a partner but not living together	171 (8%)	80 (8%)	91 (9%)
Confidence^a^, median score (quartiles)	6 (5;8)	6 (5;8)	6 (5;8)
Importance^a^, median score (quartiles)	10 (9;10)	10 (9;10)	10 (9;10)
Knowledge^a^, median score (quartiles)	5 (2;7)	5 (2;7)	5 (2;6)

^a^Single item with 1 to 10 response options; please see Additional file 2 for full details

Among those who were allocated to the intervention group, there were 144 participants who asked not to receive any more text messages before the 4-month intervention period had passed. We did not investigate further the reasons why these individuals decided not to receive any more messages (in line with the consent given). This means, however, that 86.5% (*n* = 919) of participants used the support tool for the 4-month study period and received text messages throughout the period with supportive content. The mean number of weekly screens was 5.6 among intervention group participants, and 42% used the goal setting module to set at least one goal. Among those who set at least one goal, the mean number of goals set was 3.5. Furthermore, more than 739 personal reminder messages were authored by intervention group participants. When evaluating the intervention at the 4-month follow-up interval using the System Usability Scale, the mean score was 82 out of 100 among those who responded to the questionnaire (*n* = 480, 45%). Scores above 80 are generally considered to indicate high usability and that there are no major issues using the application [38].

Among those who were randomised to the control conditions (Info-1 and Info-2), the rate at which participants clicked on the supplied link was similar: 49% in Info-1 and 51% in Info-2 (OR = 1.07, 95% CI = 0.85;1.37, probability of effect = 73.3%, *P*-value = 0.54).

All individuals were followed up, including those who stopped the intervention and those who did not click on the link. At the 2-month follow-up interval, primary outcome measures were collected from 73% of randomised participants, and at the 4-month follow-up interval, 67% of randomised participants reported on outcome measures. Follow-up data was successfully collected by phone at 2 months for 219 participants in the intervention group and 157 in the control group and at 4 months for 184 participants in the intervention group and 136 in the control group. Participants with data available were included in complete-case analyses, and all randomised participants were included in imputed analyses.

**Fig. 3:**
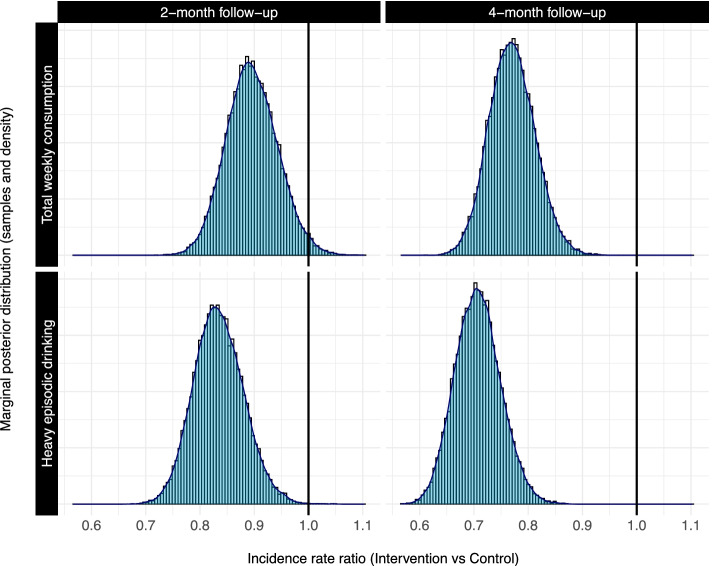
Posterior distributions of effects on alcohol consumption outcomes at 2 and 4 months comparing allocation to the digital alcohol intervention versus alcohol information control

Outcome measures and Bayesian estimates of effects given by adjusted regression models are presented in Table 2. Both complete-case analyses and sensitivity analyses with missing data imputed are presented, with no marked difference in estimates. Figure 3 shows the posterior distribution of effects for the two primary outcomes at both 2 and 4 months. As is evident in the figures, the distributions are all shifted to the left of the null, suggesting evidence of a positive effect on alcohol consumption. Unadjusted models were also estimated (please see Additional file 5: Table S1). Findings were no different from those of the adjusted models.

**Table 2:** Primary and secondary outcome measures at 2- and 4-month follow-ups and effect estimates comparing the digital intervention and alcohol information groups — adjusted models

		Adjusted regression^a^					
		Complete case^b^			Imputed		
Intervention	Control	Estimate^c^ (95% CI)	Probability of effect	***P***-value	Estimate^c^ (95% CI)	Probability of effect	***P***-value
**2-month follow-up**							
Total weekly alcohol consumption, median (quartiles), mean (sd)							
7 (3;12) 9.4 (9.7)	8 (3;15) 10.5 (10.5)	0.89 (0.81;0.99)	98.2%	0.033	0.90 (0.82;1.00)	97.8%	0.043
Frequency of heavy episodic drinking, median (quartiles), mean (sd)							
2 (1;5) 3.8 (5.2)	3 (1;7) 4.7 (5.8)	0.83 (0.75;0.93)	> 99.9%	0.0009	0.84 (0.75;0.94)	99.9%	0.0016
Risky drinking, *n* (%)							
613 (79.3%)	634 (81.7%)	0.85 (0.66;1.09)	89.4%	0.21	0.84 (0.65;1.08)	91.3%	0.17
**4-month follow-up**							
Total weekly alcohol consumption, median (quartiles), mean (sd)							
6 (2;12) 8.5 (9.1)	8 (4;15) 11.0 (10.4)	0.77 (0.69;0.86)	> 99.9%	< 0.0001	0.79 (0.72;0.88)	> 99.9%	< 0.0001
Frequency of heavy episodic drinking, median (quartiles)							
2 (0;4) 3.3 (4.9)	3 (1;6) 5.0 (6.3)	0.71 (0.63;0.79)	> 99.9%	< 0.0001	0.73 (0.65;0.82)	> 99.9%	< 0.0001
Risky drinking, *n* (%)							
523 (75.6%)	619 (84.4%)	0.58 (0.44;0.76)	> 99.9%	< 0.0001	0.59 (0.45;0.76)	> 99.9%	< 0.0001

^a^Negative binomial regression for total weekly alcohol consumption and frequency of heavy episodic drinking; logistic regression for risky drinking. Regression models adjusted for baseline values of the respective primary outcome, sex, civil status, age, motivation, importance, and knowledge

^b^*Two-month complete-case*: total weekly consumption *n* = 1557, frequency of heavy episodic drinking *n* = 1548, risky drinking *n* = 1548. *Four-month complete case*: total weekly consumption *n* = 1429, frequency of heavy episodic drinking *n* = 1424, risky drinking *n* = 1424. *All imputed analyses*: all randomised participants (*n* = 2129)

^c^Marginal posterior incidence rate ratios (IRRs) for total weekly alcohol consumption and frequency of heavy episodic drinking; marginal posterior odds ratios (ORs) for risky drinking

Total weekly alcohol consumption was lower in the intervention group than in the control group at both the 2- and 4-month follow-up intervals. Estimated incidence rate ratios (IRRs) indicated that the intervention group reported drinking 89% of the control group amount at 2 months and 77% at 4 months. The evidence was in strong favour of a positive effect on this outcome, with a 98.2% probability of effect at 2 months and > 99.9% probability of effect at 4 months. Null hypothesis tests yielded *P*-values of 0.033 at 2 months and < 0.0001 at 4 months.

The frequency of heavy episodic drinking was also less in the intervention group than in the control group at both the 2- and 4-month follow-up intervals. Estimated IRRs suggested that the intervention group was drinking heavily approximately 1 in 6 fewer occasions (or 83% as often) as the control group at 2 months and approximately 1 in 4 fewer occasions (or 71%) at 4 months. The evidence was again in strong favour of a positive effect at both intervals (probability of effect > 99.9% at both intervals; *P*-value = 0.0009 at 2 months and < 0.0001 at 4 months).

Finally, the odds of risky drinking in the intervention group were estimated to be 0.85 times that in the control group at 2 months and 0.58 times that in the control group at 4 months. The probability of an effect was slightly lower at 2 months in comparison to other outcomes (probability of effect = 89.4%, *P*-value = 0.21) but was again high at 4 months (probability of effect > 99.9%, *P*-value < 0.0001).

There was no marked difference in primary outcomes with respect to the two informed consent layouts nor with respect to the two control conditions. For details, please see Additional file 5: Table S2 and Table S3.

There was evidence of an interaction between group allocation and age on total weekly consumption only at 4 months (IRR = 0.99, 95% CI = 0.98;1.00; probability of association = 99.5%, *P*-value = 0.0066), suggesting that the intervention was more effective the older the participant. There was also evidence of an interaction between response to the baseline confidence item and group allocation on both total weekly consumption at 4 months (IRR = 0.94, 95% CI = 0.90;0.98; probability of association = 99.9%, *P*-value = 0.0019) and frequency of heavy episodic drinking at 4 months (IRR = 0.95; 95% CI = 0.91;0.99, probability of association = 99.0%, *P-*value = 0.019). Again, this suggests that the intervention was more effective among those who were more confident about their ability to reduce their drinking at study entry. Finally, the effect of the intervention on the frequency of heavy episodic drinking at 4 months also varied by baseline frequency of heavy episodic drinking (IRR = 0.98, 95% CI = 0.97;1.00, probability of association = 98.9%, *P*-value = 0.014), suggesting that the intervention was more effective among more frequent heavy drinkers. Estimates of effects in multi-level models, with random intercept and slope for age, were unchanged from the primary adjusted models (and not superior with respect to WAIC). There was no strong evidence for moderation of effects at the 2-month interval.

There was evidence that older individuals, and individuals who were less frequent heavy drinkers at baseline, were more likely to respond to both the 2- and 4-month follow-up (please see Additional file 6 for full details). Group allocation was not observed to be markedly associated with missingness at the 2-month follow-up (OR = 0.99; 95% CI = 0.93;1.09, probability of association = 53.6%) nor at the 4-month follow-up (OR = 1.03; 95% CI = 0.97;1.30, probability of association = 78.5%). We post hoc added further exploration of moderating effects between baseline characteristics and group allocation on missingness and found only weak evidence of an interaction between baseline heavy episodic drinking and group allocation. Please see Additional file 6 for full details of these attrition analyses. There were no marked patterns of association between follow-up attempts and any of the primary outcomes at any of the follow-up intervals.

## Discussion

We found strong evidence that a digital alcohol intervention designed around weekly self-monitoring, with feedback and tools for behaviour change, helped reduce both self-reported total alcohol consumption and frequency of heavy episodic drinking outcomes among people seeking help online, in comparison to generally available online alcohol information. The outcomes indicate that effects were observed after 2 months, and these strengthened after 4 months. There was no later follow-up study, and the longer-term durability of the effects is thus unknown.

The recruitment procedures, intervention, and study design closely follow what might be expected of non-research implementation of this intervention. This study is thus to be interpreted as an effectiveness trial [39]. Participants found the study when searching for help online, they signed up and used the intervention without any personal guidance, and they found it attractive to use. Therefore, the gap between what was done in the trial and what could be done in a broader dissemination of the intervention is small. The generalisability of our findings appears strong, behoving forensic scrutiny of study limitations. Broader dissemination may include recommending the intervention on websites dedicated to alcohol risk reduction, cancer awareness and prevention websites, social media campaigns by the national alcohol monopoly, and recommendation by health care professionals in primary health care.

### Limitations

Attrition was a prominent concern in designing the trial, and it was somewhat higher than anticipated. We assumed that it would be in the range of 5 to 25%, despite being intrinsically challenging in online trials with minimal barriers to participation [40]. We found, however, it was approximately 27% at the 2-month follow-up and 33% at the 4-month follow-up. Analyses of baseline characteristics revealed that age and frequency of heavy episodic drinking at baseline were potentially associated with loss to follow-up, though there was only weak evidence that the latter was differential between groups. Group allocation in and of itself was not markedly associated with missingness. Both age and heavy episodic drinking at baseline were included as predictors when imputing values, and the imputed analyses did not result in any changes of our findings. Nonetheless, most conservatively, the study findings may be interpreted as most secure for older less frequent drinkers.

A second component of the power calculation which did not meet our expectations was the magnitude of effect. We anticipated a difference in alcohol consumption around 15%, which determined the sample size of 2126; however, as the effect estimate was almost twice this, we ended up recruiting more participants than necessary to draw our conclusions. Pre-specifying sample sizes using traditional power calculations have been raised as an ethical concern [41], as it may both over- and under-recruit participants. Future trials of a similar character to the current trial should consider using Bayesian designs which allow for using criteria which are continuously evaluated as data is collected, avoiding a fixed sample size which may result in too few or too many participants recruited [42–44].

We told participants in the control group that they were being given information about alcohol and health as motivation to think more about reducing their alcohol consumption and that they later would also receive additional support through their mobile phone. This was an attempt to convey to control participants that this was one way the support was intended to work, rather than saying that they had to wait for support. However, it is unavoidable that this can be perceived to a greater or lesser extent as being asked to wait for the support tool. Therefore, concerns about possible biases in waiting list designs arising out of disappointment cannot be ruled out [28, 45].

The previous concern ties into other possible sources of bias that were a feature of this trial, which were lack of blinding of participants and reliance for outcome ascertainment on self-reported behavioural data. The conduct of an effectiveness trial militates against the blinding of participants. In an efficacy trial context, we may have considered some elements of deception [46], for example constructing a pseudo-intervention control condition. Instead, we created a control condition which incorporated the kinds of alcohol health information that people will encounter when searching online, as a variant on a treatment-as-usual design, i.e. what would occur on the absence of our intervention [27].

These are common threats to valid inference in trials of digital alcohol interventions [8, 9] and other kinds of online trials [47]. We need to find ways of addressing these kinds of issues in future trials, preferably by design, e.g. by introducing blinding or using factorial trials, or by accounting for possible biases in the analysis [25] if design solutions are not viable.

Relatedly, although dedicated randomised studies examining the validity of online self-reported drinking behavioural data are reassuring [48], the operation of social desirability biases on self-report in these kinds of studies remains not well understood. It is entirely plausible that those waiting for the intervention not only wait to reduce their drinking, but are more likely to report not having reduced their drinking for impression management reasons [49]. Similarly, those having received the intervention may be more likely to feel implicitly that they should report drinking less [50]. In addition, to reduce participant burden, we used a summary measure for weekly consumption which may be considered less valid than a timeline follow-back method, though there is no other reason to suppose that any measurement bias will be differential. Even small biases in self-reported outcomes are particularly important in trials where the intervention effects are also small, as here [51].

This trial saw a decline in self-reported drinking in both groups from baseline to follow-up. In trials of alcohol interventions where participants are eligible if they have been screened as excessive drinkers, we expect alcohol consumption to be lower in both groups at follow-up, and this is commonly the case [52–55]. This difference over time is partially due to regression to the mean, as a consequence of not including non-risky drinkers [52, 53]. It is also possible that participants’ behaviour within this trial was affected by finding the trial online, signing up, baseline assessment, knowledge of participation, and other research participation effects [54]. Interpretation of pre-post differences within groups is therefore challenging, being strongly confounded by external factors, which is exactly what the randomised comparison protects against. Differences between groups at follow-up therefore provide the valid estimate of effect [55], i.e. the relative difference in outcomes between two groups which were comparable before treatment assignment.

The two follow-up intervals of 2 and 4 months prohibit any further examination of long-term effects of the intervention on alcohol consumption. In designing the study, it was anticipated that attrition would drop further after 4 months, as participants were members of the public who found the study and signed up by their own volition, i.e. without any active recruitment process. We judged it preferable to avoid adding a third longer follow-up period knowing that attrition would grow and estimates of effects more likely to be biased by attrition. It should be noted, however, that the digital intervention which was studied has been designed to be used by participants for as long as they prefer, meaning that continued support can be given for those who want it. The 4-month cut-off was thus due to study constraints rather than the digital intervention having a fixed duration of exposure.

The trial included two sub-studies concerning presentation of consent materials and control group information. While these could potentially have affected the outcome of the main study, we found no strong evidence of any difference in outcomes with respect to these two random allocations (please see Additional file 5). We therefore believe that any interaction between the sub-studies and the main trial is negligible with respect to trial findings.

Finally, while study procedures were automated and research personnel were blinded, there was a risk of observer bias when calling participants for follow-up data. The person responsible for calling to collect follow-up data (KÅ) was not aware of allocation and did not ask about anything other than the two primary outcome measures, yet participants did on occasion reveal that they had or had not received the digital intervention. However, in almost all cases, this was after responses were collected, which means that while the magnitude of this bias can be large, in this study, the risk is likely to be small.

### Research in context

The findings of this study are regarded as strong, within the paradigm of conventional online trial designs and subject to the range of study limitations stringently considered here. The size of the observed effects thus warrants some scrutiny. While our adjusted relative effect estimates are not directly comparable to the absolute differences estimated in meta-analyses, inspecting the differences in mean total weekly alcohol consumption in Table 2 suggests that the intervention group was drinking 30 g less at 4 months (Cohen’s *d* = 0.25). This compares with meta-analytic estimates of effects of 32.3 g (mean follow-up interval 3.4 months) [7], 22.8 g (mean follow-up interval 5.5 months) [8], and 18.6 g (mean follow-up interval 3.1 months) [9]. Moreover, synthesised effects of frequency of heavy episodic drinking have suggested that digital interventions may reduce the expected number of episodes per month by 0.24 (mean follow-up interval 3.3 months) [8] and 0.33 (mean follow-up interval 3.1 months) [9], whilst in this trial, we observed a difference in means of 1.7 episodes per month (Cohen’s *d* = 0.29). Due to retention in online trials being a challenge, the trial did not investigate follow-up after 4 months, which prohibits consideration of the existence of any longer-term effects.

## Conclusions

A digital alcohol intervention produced self-reported behaviour change among online help seekers in the general population. These findings are encouraging, and this study contributes to the global literature in numerous ways. No suggestion is made that intervening with individuals in the general population by itself is a means of reducing alcohol harms in society; for this, evidence-informed alcohol policies which shift the entire distribution of alcohol consumption are needed [10, 56]. This study further demonstrates, however, that one component of the societal response can be offering help online to people who seek it and that simple digital tools centred on weekly monitoring can be valued and can make a difference to alcohol consumption.

## Supplementary Information

**Additional file 1.** Informed consent materials.

**Additional file 2.** Baseline questionnaire.

**Additional file 3.** Content of digital intervention.

**Additional file 4.** Allocation text messages.

**Additional file 5. **Supplementary analyses. Tables S1-S3. **Table S1**. Primary and secondary outcome measures at 2- and 4-month follow-ups and effect estimates comparing the digital intervention and alcohol information groups – unadjusted models. **Table S2**. Primary outcomes measured at 2- and 4-month follow-ups and effect estimates comparing Consent-1 and Consent-2 groups – adjusted models. **Table S3**. Primary outcomes measured at 2- and 4-month follow-ups and effect estimates comparing Info-1 and Info-2 groups – adjusted models.

**Additional file 6. **Attrition analyses. Figures S1-S2. **Figure S1**. Baseline age versus response/no-response at 2- and 4-months stratified by allocated group. **Figure S2**. Baseline frequency of heavy episodic drinking versus response/no-response at 2- and 4-months stratified by allocated group.

## Data Availability

A study protocol, including a statistical analysis plan, is available open-access [17]. Deidentified datasets generated during and/or analysed during the current study will be made available upon reasonable request to the corresponding author, after approval of a proposal and with a signed data access agreement.

## Abbreviations

CI: Confidence/credible interval
DALY: Disability-adjusted life year
IRR: Incidence rate ratio
MLE: Maximum likelihood estimation
OR: Odds ratio
SD: Standard deviation
WAIC: Widely Applicable Information Criterion
