# Supplementary material for: Effectiveness of a digital intervention versus alcohol information for online help-seekers in Sweden: a randomised controlled trial
Source: BMC Med. 2022 May 17;20:176. doi: 10.1186/s12916-022-02374-5 (PMC9112593; doi:10.1186/s12916-022-02374-5)
Supplement: Supplementary file 1 — Additional file 1. Informed consent materials. [file 12916_2022_2374_MOESM1_ESM.pdf]

EFFECTIVENESS OF A DIGITAL INTERVENTION VERSUS ALCOHOL INFORMATION FOR ONLINE HELP-SEEKERS IN SWEDEN: A RANDOMISED CONTROLLED TRIAL

ADDITIONAL FILE 1

INFORMED CONSENT MATERIALS

All individuals registering interest will be given information about the trial procedures prior to giving informed consent. Individuals will be randomised to Consent-1 or Consent-2 which differ in the way that the information is presented and structured. Both versions will begin with a general introduction and information about allocation to intervention and control, followed by information about how personal data is handled, and finally how data is to be analysed and reported. However, the two latter parts of the consent information will be made available through clickable hyperlinks for Consent-2.

Consent-1

Below you will find information about the study that you should read before you consent to participating. You consent by pressing the button “I have read and give consent”. If you agree to participate in the trial, we will first ask you a series of questions.

This trial aims to estimate the effect of using a novel support tool on alcohol consumption among risky drinkers in the general population of Sweden. The tool has been developed by researchers at Linköping University. Using a mobile phone, users are asked to assess their consumption on a weekly basis and are given access to a dashboard which they can use to track their consumption, learn more about how to reduce consumption levels, and why it is important to make sure that one does not drink too much. Users will also receive motivating SMS messages throughout the week. There are no costs involved for the user. Users can at any time turn off the tool by responding to one of the SMS with the word STOP.

In order to investigate the effect of the intervention we need to compare two groups of individuals. Therefore, all individuals that agree to participate in the study will randomly be allocated into one of two groups. One group will begin the trial by being given information that will motivate them to reduce their alcohol consumption for four months, and then have access to the new support tool for four months. The other group will have these two phases reversed, thus being given immediate access to the new support tool. Regardless of which group you are allocated to, we will contact you in one, two and four months to ask you a series of questions.

<u>Information about personal information</u>

If you decide to join the study, personal information about you will be collected and stored during the project. The purpose of the data collection is research, and public interest therefore forms a legal basis for the collection of personal information. Your responses to the baseline and follow-up questionnaires will be recorded. Your interactions with the support tool will also be stored for analysis. All your data will be connected to an encrypted version of your phone number, which means that it is necessary to know a secret key in order to identify which responses belong to which phone number. This secret key will only be known to the primary investigator of this research project (Marcus Bendtsen). When the project is complete, all phone numbers will be deleted, which means that it is no longer possible to trace an individual’s data.

Your data will be treated with confidentiality. Linköping University is responsible for your personal data. According to EU law (GDPR EU 2016/679) you have the right to access your personal data free of charge, and you are allowed to ask for changes to be made if the data is erroneous. You can also request that all your personal data be erased. Deletion of data will be done if it is deemed that it will not change the outcome of the trial. If you want to access your data you should email the primary investigator (marcus.bendtsen@liu.se). The ombudsman can be contacted at dataskyddsombud@liu.se. If you have complaints about how your personal data has been treated you should contact Datainspektionen.

Collection of data, and sending of SMS messages, will be handled by AlexIT AB, which is owned and run by the primary investigator (Marcus Bendtsen). Only the primary investigator (Marcus Bendtsen) will have access to the secret key used to encrypt phone numbers. Please note that once the data collection has been finalised the secret key and all phone numbers will be erased, and it will no longer be possible to trace an individual phone number to a response. All data will be removed from AlexIT AB and a final copy of the data (without phone numbers) will be stored at Linköping University. This means that the data stored is no longer considered personal data, and that it is no longer possible to request access nor removal of data. There is a signed agreement between Linköping University and AlexIT AB that states that AlexIT AB is allowed to help with the collection of data for research projects conducted at Linköping University (dnr IMH-2018-00351).

In summary, after the project is completed only anonymous data will be stored. This means that it will not be possible to trace data to specific individuals, and it is therefore not possible to find your specific data on request.

<u>Information about how data will be analysed and results reported</u>

Data collected will be analysed at group level, thus no individual responses will be traceable through the analysis. To estimate the effect, and measure whether or not the effect is significant, we will create so called regression models. These models tell us how alcohol consumption differs between those who first received the support tool and those who did not. The difference is indicative of the effect of the intervention. These models will then form a basis for a scientific publication that will contain an explanation of the trial procedure and the results that we have found.

Consent-2

Below you will find information about the study that you should read before you consent to participating. You consent by pressing the button “I have read and give consent”. If you agree to participate in the trial, we will first ask you a series of questions.

This trial aims to estimate the effect of using a novel support tool on alcohol consumption among risky drinkers in the general population of Sweden. The tool has been developed by researchers at Linköping University. Using a mobile phone, users are asked to assess their consumption on a weekly basis and are given access to a dashboard which they can use to track their consumption, learn more about how to reduce consumption levels, and why it is important to make sure that one does not drink too much. Users will also receive motivating SMS messages throughout the week. There are no costs involved for the user. Users can at any time turn off the tool by responding to one of the SMS with the word STOP.

In order to investigate the effect of the intervention we need to compare two groups of individuals. Therefore, all individuals that agree to participate in the study will randomly be allocated into one of two groups. One group will begin the trial by being given information that will motivate them to reduce their alcohol consumption for four months, and then have access to the new support tool for four months. The other group will have these two phases reversed, thus being given immediate access to the new support tool. Regardless of which group you are allocated to, we will contact you in one, two and four months to ask you a series of questions.

For more information about how personal data is handled, please click here: LINK (which will show the same information as Consent-1)

For more information about how data is analysed and results reported, please click here: LINK (which will show the same information as Consent-1).
