# Supplementary material for: Effectiveness of a digital intervention versus alcohol information for online help-seekers in Sweden: a randomised controlled trial
Source: BMC Med. 2022 May 17;20:176. doi: 10.1186/s12916-022-02374-5 (PMC9112593; doi:10.1186/s12916-022-02374-5)
Supplement: Supplementary file 2 — Additional file 2. Baseline questionnaire. [file 12916_2022_2374_MOESM2_ESM.pdf]

EFFECTIVENESS OF A DIGITAL INTERVENTION VERSUS ALCOHOL INFORMATION FOR ONLINE HELP-SEEKERS IN SWEDEN: A RANDOMISED CONTROLLED TRIAL

ADDITIONAL FILE 2

BASELINE QUESTIONNAIRE

1. Sex:
    a. Female
    b. Male

2. Civil status:
    a. Living alone without kids at home
    b. Living alone with kids at home
    c. Living with somebody without kids
    d. Living with somebody with kids
    e. Have a partner but not living together

3. Age (numerical measure)

4. How many standard drinks did you consume last week? (numerical measure)

5. How often, during the past month, have you consumed four/five (female/male) or more standard drinks on one occasion? (numerical measure)

6. How confident are you that you will be able to reduce your alcohol consumption? (10-point scale ranging from 1 = “Not at all” to 10 = “Very confident”)

7. How important is it for you to reduce your alcohol consumption? (10-point scale ranging from 1 = “Not important” to 10 = “Very important”)

8. How well do you know how to reduce your alcohol consumption? (10-point scale ranging from 1 = “Not well at all” to 10 = “Very well”)

Note: Participants are reminded of the definition of a standard drink by graphical means.
