# Supplementary material for: Effectiveness of a digital intervention versus alcohol information for online help-seekers in Sweden: a randomised controlled trial
Source: BMC Med. 2022 May 17;20:176. doi: 10.1186/s12916-022-02374-5 (PMC9112593; doi:10.1186/s12916-022-02374-5)
Supplement: Supplementary file 3 — Additional file 3. Content of digital intervention. [file 12916_2022_2374_MOESM3_ESM.pdf]

EFFECTIVENESS OF A DIGITAL INTERVENTION VERSUS ALCOHOL INFORMATION FOR ONLINE HELP-SEEKERS IN SWEDEN: A RANDOMISED CONTROLLED TRIAL

ADDITIONAL FILE 3

CONTENT OF DIGITAL INTERVENTION

The digital intervention aimed to target improving motivation and self-efficacy, as well as teaching new skills and addressing environmental constraints - which are understood to improve the likelihood of successful behaviour change, including for changing one's drinking.[21] Although the evidence is not yet strong on how to best manipulate the aforementioned components with respect to alcohol consumption, promising actions to promote include those that focus on behaviour substitution, problem solving, goal setting, review of behavioural goals, self-monitoring, and normative feedback.[22,23] Thus, modules included in the digital intervention revolved around these activities.

The core element of the digital intervention was a text message sent to participants each Sunday afternoon. The text message included a prompt to self-monitor one's current alcohol consumption, and a hyperlink to a web-based screening tool. Those who decided to click on the link were first screened with respect to total alcohol consumption in the past week, and frequency of episodes of heavy drinking the past month. Thereafter, they were given access to a personalised support tool which consisted of six modules contained in a web-based dashboard. The modules were:

1. Normative comparison of the participants' current consumption compared with others of the same age group and gender (based on data from Sweden), and risky drinking classification.

2. Information about some of the risks from drinking alcohol, including risk of disease, how it may affect children in proximity, driving, and other negative consequences. This module also allowed participants to simulate how different levels of consumption affected risk of cardiovascular disease.

3. One module allowed participants to create a plan which they could use when facing an environmental or behavioural trigger (e.g., going to the pub). This module asked participants to write a text message to themselves and pick a time and date for when they wanted to receive this message in the coming week (up to 3 times).

4. General tips to strengthen participants' know-how on how to reduce their consumption was given in one module. The tips included suggestions to create prompts or cues as reminders that they have committed to reduce their drinking, as well as suggesting that participants practice a new behaviour and substitute their current behaviour with a different one (e.g., replacing at least two alcoholic beverages with non-alcoholic beverages each week). The tips also concerned identification of relapse triggers and barriers, avoiding social cues for drinking, and environmental restructuring (e.g., avoid keeping alcohol at home).

5. One module showed participants' consumption over time (with data from the weekly assessments). Participants could set a goal for their consumption, which would then show up graphically in the

chart. This allowed participants to set and review their own goals while also visualising the discrepancy between their current consumption and their goals.

6. On Wednesdays, Fridays, and Saturdays, participants received additional text messages with content aimed to increase motivation and skills. Participants could also opt for additional text messages to be sent on the Mondays, Tuesdays, and Thursdays. The messages sent were a refinement of a previously developed set that was created through formative development and reported previously.[24]
