# Supplementary material for: Effectiveness of a digital intervention versus alcohol information for online help-seekers in Sweden: a randomised controlled trial
Source: BMC Med. 2022 May 17;20:176. doi: 10.1186/s12916-022-02374-5 (PMC9112593; doi:10.1186/s12916-022-02374-5)
Supplement: Supplementary file 4 — Additional file 4. Allocation text messages. [file 12916_2022_2374_MOESM4_ESM.pdf]

EFFECTIVENESS OF A DIGITAL INTERVENTION VERSUS ALCOHOL INFORMATION FOR ONLINE HELP-SEEKERS IN SWEDEN: A RANDOMISED CONTROLLED TRIAL

ADDITIONAL FILE 4

ALLOCATION TEXT MESSAGES

Participants allocated to the intervention group will receive a text message with the following content (note: if a participant signs up on a Sunday, then "You will receive your first text on Sunday" will be replaced with "You will soon receive your first text"):

You have been allocated to the group that will be given immediate access to the mobile phone support tool. "You will receive your first text on Sunday"/"You will soon receive your first text". Until then, we recommend that you read about alcohol, health and society here: www.iq.se.

Participants allocated to the control group will receive a text message with the following content:

You have been allocated to the group that will begin by accessing information that will help motivate you to think more about reducing your alcohol consumption. After this period of time you will receive an additional mobile phone support tool. The other group will have these phases reversed. In one, two and four months from now we will send you questionnaires. Your responses to these questionnaires will help us understand better how we can help individuals who wish to reduce their alcohol consumption.

You will soon receive a text messages containing information about the short and long term consequences of alcohol consumption.

Shortly thereafter they will receive one out of two messages depending on group allocation (Info-1 and Info-2).

<u>Info-1 (Brief information emphasising complexity from industry sources)</u>
Alcohol poses both short-term and long-term risks to your health and well-being.

There is obvious evidence of an association between alcohol consumption and violence, however the relationship between alcohol and violence or anti-social behaviour is highly complex.

It is simply not true to describe violence and crime as 'alcohol fuelled'. Alcohol does not fuel violence. If it did, every drinker would become angry or violent when sufficiently intoxicated.

International evidence demonstrates increased risk of some cancers is most significantly associated with heavy drinking patterns. However, the relationship between cancer risk and light to moderate drinking appears more complex.

For more information on alcohol, health and society, go to LINK

<u>Info-2 (Brief information from public health perspective)</u>
Alcohol poses both short-term and long-term risks to your health and well-being.

Alcohol causes violence and other anti-social behaviour. The more you drink, or the more frequently you get drunk, the more you risk being either a perpetrator or a victim.

This certainly does not mean that every drinker will inevitably become violent. Alcohol reduces inhibitions and violence or other anti-social behaviour are possible consequences.

International evidence demonstrates increased risk of a range of cancers at all levels of drinking. Even at low levels. The more you drink the greater the risk of cancer over the long term.

For more information on alcohol, health and society, go to LINK
