# Supplementary material for: Effectiveness of a digital intervention versus alcohol information for online help-seekers in Sweden: a randomised controlled trial
Source: BMC Med. 2022 May 17;20:176. doi: 10.1186/s12916-022-02374-5 (PMC9112593; doi:10.1186/s12916-022-02374-5)
Supplement: Supplementary file 5 — Additional file 5. Supplementary analyses. Tables S1-S3. Table S1. Primary and secondary outcome measures at 2- and 4-month follow-ups and effect estimates comparing the digital intervention and alcohol information groups – unadjusted models. Table S2. Primary outcomes measured at 2- and 4-month follow-ups and effect estimates comparing Consent-1 and Consent-2 groups – adjusted models. Table S3. Primary outcomes measured at 2- and 4-month follow-ups and effect estimates comparing Info-1 and Info-2 groups – adjusted models. [file 12916_2022_2374_MOESM5_ESM.pdf]

EFFECTIVENESS OF A DIGITAL INTERVENTION VERSUS ALCOHOL INFORMATION FOR ONLINE HELP-SEEKERS IN SWEDEN: A RANDOMISED CONTROLLED TRIAL

ADDITIONAL FILE 5

SUPPLEMENTARY ANALYSES

Outcome measures and Bayesian estimates of intervention versus control effects given by unadjusted regression models are presented in Table S1. As pointed out in the main text, these were not markedly different from the primary adjusted models. Table S2 presents outcome measures and Bayesian estimates of intervention Consent-1 and Consent-2 effects. As can be seen by the probability of effect estimates, there was no strong evidence of an effect in either direction. Likewise, when comparing Info-1 versus Info-2, there was no strong evidence of an effect in either direction (Table S3).

Table S1 - Primary and secondary outcome measures at 2- and 4-month follow-ups and effect estimates comparing the digital intervention and alcohol information groups – unadjusted models

		Unadjusted regression[a]		
		Complete case[b]		
**Intervention**	**Control**	**Estimate[c] (95% CI)**	**Probability of effect**	**P-value**
**2-month follow-up**				
Total weekly alcohol consumption, median (quartiles)				
7 (3;12)	8 (3;15)	0.89 (0.80;1.00)	97.9%	0.041
Frequency of heavy episodic drinking, median (quartiles)				
2 (1;5)	3 (1;7)	0.80 (0.71;0.90)	> 99.9%	0.0002
Risky drinking, n (%)				
613 (79.3%)	634 (81.7%)	0.86 (0.67;1.11)	88.0%	0.23
**4-month follow-up**				
Total weekly alcohol consumption, median (quartiles)				
6 (2;12)	8 (4;15)	0.77 (0.69;0.86)	> 99.9%	< 0.0001
Frequency of heavy episodic drinking, median (quartiles)				
2 (0;4)	3 (1;6)	0.66 (0.58;0.75)	> 99.9%	< 0.0001
Risky drinking, n (%)				
523 (75.6%)	619 (84.4%)	0.58 (0.45;0.74)	> 99.9%	< 0.0001

[a] Negative binomial regression for total weekly alcohol consumption; logistic regression for risky drinking.

[b] **2-month complete-case**: total weekly consumption n = 1557, frequency of heavy episodic drinking n = 1548, risky drinking n = 1548. **4-month complete case**: total weekly consumption n = 1429, frequency of heavy episodic drinking n = 1424, risky drinking n = 1424.

[c] Marginal posterior incidence rate ratios (IRRs) for total weekly alcohol consumption and frequency of heavy episodic drinking; marginal posterior odds ratios (ORs) for risky drinking.

Table S2 - Primary outcomes measured at 2- and 4-month follow-ups and effect estimates comparing Consent-1 and Consent-2 groups – adjusted models

		Adjusted regression[a]		
		Complete case[b]		
**Consent-1**	**Consent-2**	**Estimate[c] (95% CI)**	**Probability of effect**	**P-value**
**2-month follow-up**				
Total weekly alcohol consumption, median (quartiles)				
8 (3;13)	8 (3;14)	0.96 (0.87;1.06)	78.6%	0.42
Frequency of heavy episodic drinking, median (quartiles)				
2 (1;5)	2 (1;5)	0.99 (0.89;1.11)	55.5%	0.89
**4-month follow-up**				
Total weekly alcohol consumption, median (quartiles)				
7 (3;13)	8 (3;14)	0.95 (0.85;1.06)	83.4%	0.33
Frequency of heavy episodic drinking, median (quartiles)				
2 (1;5)	2 (1;5)	0.96 (0.86;1.08)	76.3%	0.47

[a] Negative binomial regression for total weekly alcohol consumption and frequency of heavy episodic drinking; logistic regression for risky drinking. Regression models adjusted for baseline values of the respective primary outcome, sex, civil status, age, motivation, importance, and knowledge.

[b] **2-month complete-case**: total weekly consumption n = 1557, frequency of heavy episodic drinking n = 1548. **4-month complete case**: total weekly consumption n = 1429, frequency of heavy episodic drinking n = 1424.

[c] Marginal posterior incidence rate ratios (IRRs) for total weekly alcohol consumption and frequency of heavy episodic drinking; marginal posterior odds ratios (ORs) for risky drinking.

Table S3 - Primary outcomes measured at 2- and 4-month follow-ups and effect estimates comparing Info-1 and Info-2 groups – adjusted models

		Adjusted regression[a]		
		Complete case[b]		
Info-1	Info-2	Estimate[c] (95% CI)	Probability of effect	P-value
**2-month follow-up**				
Total weekly alcohol consumption, median (quartiles)				
8 (4;15)	7 (3;14)	1.09 (0.94;1.26)	87.0%	0.26
Frequency of heavy episodic drinking, median (quartiles)				
3 (1;6)	3 (1;7)	0.98 (0.84;1.13)	63.0%	0.74
**4-month follow-up**				
Total weekly alcohol consumption, median (quartiles)				
9 (4;15)	8 (4;15)	1.02 (0.89;1.18)	63.4%	0.74
Frequency of heavy episodic drinking, median (quartiles)				
3 (1;6)	3 (1;5)	1.03 (0.89;1.19)	64.3%	0.71

[a] Negative binomial regression for total weekly alcohol consumption and frequency of heavy episodic drinking; logistic regression for risky drinking. Regression models adjusted for baseline values of the respective primary outcome, sex, civil status, age, motivation, importance, and knowledge.

[b] **2-month complete-case**: total weekly consumption n = 781, frequency of heavy episodic drinking n = 775. **4-month complete case**: total weekly consumption n = 736, frequency of heavy episodic drinking n = 732.

[c] Marginal posterior incidence rate ratios (IRRs) for total weekly alcohol consumption and frequency of heavy episodic drinking; marginal posterior odds ratios (ORs) for risky drinking.
