# Supplementary material for: Effectiveness of a digital intervention versus alcohol information for online help-seekers in Sweden: a randomised controlled trial
Source: BMC Med. 2022 May 17;20:176. doi: 10.1186/s12916-022-02374-5 (PMC9112593; doi:10.1186/s12916-022-02374-5)
Supplement: Supplementary file 6 — Additional file 6. Attrition analyses. Figures S1-S2. Figure S1. Baseline age versus response/no-response at 2- and 4-months stratified by allocated group. Figure S2. Baseline frequency of heavy episodic drinking versus response/no-response at 2- and 4-months stratified by allocated group. [file 12916_2022_2374_MOESM6_ESM.pdf]

EFFECTIVENESS OF A DIGITAL INTERVENTION VERSUS ALCOHOL INFORMATION FOR ONLINE HELP-SEEKERS IN SWEDEN: A RANDOMISED CONTROLLED TRIAL

ADDITIONAL FILE 6

ATTRITION ANALYSES

At the 2-month interval, age was associated with missing data (OR = 0.98, 95% CI = 0.97;0.98, probability of association > 99.9%), as was baseline frequency of heavy episodic drinking (OR = 1.03, 95% CI = 1.01; 1.04, probability of association > 99.9%). This suggests that older individuals were more likely to respond to follow-up, as were those who were less frequent heavy drinkers at baseline. At the 4-month interval, the same two characteristics were related to missingness: age (OR = 0.97, 95% CI = 0.96;0.98; probability of association > 99.9%), and frequency of heavy episodic drinking (OR = 1.03, 95% CI = 1.01; 1.04, probability of association > 99.9%). Group allocation was not observed to be markedly associated with missingness.

To explore if there were moderating effects between baseline characteristics and group allocation on missingness at both 2- and 4-months follow-up, regression models were estimated with an interaction term between group allocation and each of the baseline characteristics (using shrinkage priors).

In the interaction model at the 2-month interval, the association between age and missingness was in principle unchanged - suggesting no moderation by group. There was evidence of a slight moderation by group for the association between missingness and frequency of heavy episodic drinking at baseline (OR = 1.01, 95% CI = 1.00;1.05, probability of association = 85.7%). However, the OR was small and the probability that the OR was greater than the null was only 85.7%.

In the interaction model at the 4-month interval, the association between age and missingness was again unchanged, and the association with frequency of heavy episodic drinking was slightly moderated (OR = 1.01, 95% CI = 0.99;1.04, probability of association = 83.3%). Again, while there was some evidence of moderation with frequency of heavy episodic drinking, the magnitude was small and the probability of an association greater than the null was not high.

Caution should be taken when generalising across the entire age and heavy episodic drinking range, as the relationship is not necessarily monotonic. Nevertheless, Figures S1 and S2 visualises a monotonic relationship between missingness and age and frequency of heavy episodic drinking at the 2- and 4-month follow-up intervals. As can be seen in the figures, there is a trend in both groups that response increases with age and decreases with baseline frequency of heavy episodic drinking.

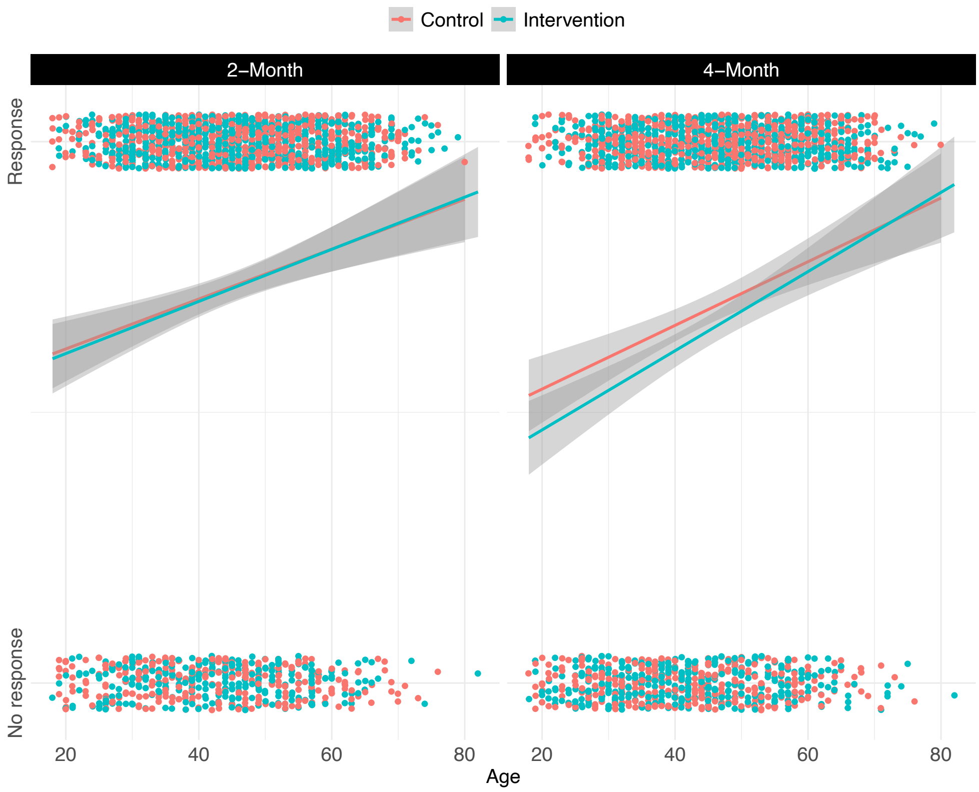

Figure S1 - Baseline age versus response/no-response at 2- and 4-months stratified by allocated group

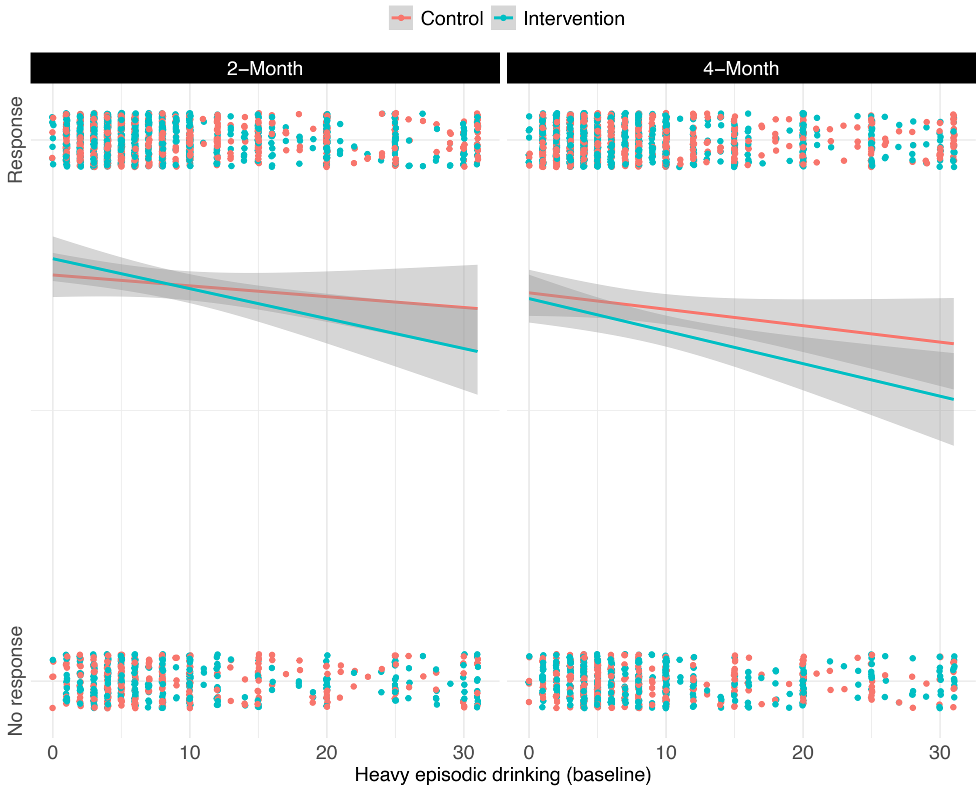

Figure S2 - Baseline frequency of heavy episodic drinking versus response/no-response at 2- and 4-months stratified by allocated group
